# Association of physical exercise characteristics with obstructive sleep apnea symptoms: A cross-sectional study

**DOI:** 10.1097/MD.0000000000049326

**Published:** 2026-06-19

**Authors:** Yan Sun, Kexin Zhu

**Affiliations:** a School of Police Combat Training, People’s Public Security University of China, Beijing, People’s Republic of China; b School of Physical Education (Main Campus), Zhengzhou University, Zhengzhou, Henan, People’s Republic of China.

**Keywords:** exercise duration, exercise intensity, metabolic equivalent, obstructive sleep apnea, physical exercise

## Abstract

Obstructive sleep apnea (OSA) increases the risk of multiple chronic diseases such as diabetes and cardiovascular disease, posing a substantial threat to public health. Therefore, this study aimed to investigate the association between physical exercise and OSA symptoms. A representative sample of 7180 individuals aged 20 years and older was selected from the 2015 to 2018 United States National Health and Nutrition Examination Survey. Binary logistic regression was used to analyze the association between physical activity and OSA symptoms. Threshold effect analysis and restricted cubic spline curves were applied to evaluate the dose-response relationships of exercise duration and metabolic equivalents of tasks (METs) with OSA symptoms. After adjustment for all covariates in the multivariable analysis, exercise was significantly inversely associated with OSA symptoms (odds ratio [OR] = 0.84, 95% confidence interval [CI]: 0.76–0.93, *P* < .05). Compared with non-exercisers, moderate-intensity exercisers had OR = 0.89 (95% CI: 0.79–1.00, *P* = .059), while high-intensity exercisers showed a stronger association (OR = 0.79, 95% CI: 0.69–0.90, *P* < .05). Threshold analyses suggested potential statistical inflection points at approximately 75 minutes of exercise and 320 METs (*P* < .05). Physical activity was inversely associated with OSA symptoms, with high-intensity exercise associated with a relatively lower likelihood of OSA symptoms. The association between exercise and OSA symptoms showed a potential change near 75 minutes of exercise and 320 METs. Future population-based intervention studies should assess the clinical application of these activity levels in improving OSA outcomes.

## 1. Introduction

Obstructive sleep apnea (OSA) symptoms is a prevalent sleep-disordered breathing condition characterized by recurrent collapse and obstruction of the upper airway during sleep, leading to decreased oxygen saturation and disrupted sleep architecture.^[1]^ Clinical manifestations include loud and irregular snoring at night, apnea-induced awakenings, morning headaches, dry mouth, daytime drowsiness, and fatigue.^[2]^ Chronic hypoxemia can easily trigger hypertension and increase the risks of coronary heart disease and cardiac arrhythmias, potentially leading to myocardial infarction or sudden death in severe cases.^[3,4]^ Disrupted sleep architecture and hypoxia deprive the brain of adequate rest, resulting in memory impairment, poor concentration, and an increased risk of dementia.^[5,6]^ OSA also affects glucose and lipid metabolism, leading to insulin resistance, exacerbating obesity, and increasing the risks of developing diabetes mellitus, hyperlipidemia, and other related diseases.^[7–9]^ Given its detrimental impact on human health, identifying factors associated with OSA symptoms is of critical importance.

Previous studies have indicated that obese individuals are at a higher risk of developing OSA symptoms. Weight gain and fat deposition, particularly around the neck and upper airway, reduce airway patency and increase collapsibility.^[10,11]^ Excessive central fat deposition can lead to a reduction in lung capacity, which may subsequently decrease longitudinal tracheal traction and pharyngeal wall tension, thereby altering the “tube law” of the pharyngeal airway (the lung-volume dependency of the upper airway).^[12]^ Additionally, neck circumference can serve as a predictor of OSA symptoms and its severity.^[13]^ Physical activity may help reduce the risk of overweight and obesity^[14]^ and could contribute to maintaining muscle mass, including respiratory muscle function, and may limit excess fat accumulation.^[15,16]^ In addition, sedentary behavior has been associated with a higher likelihood of OSA symptoms.^[17]^

However, the association of physical activity (particularly exercise duration and intensity) with OSA symptoms remains unclear. In addition, most prior studies have focused on overall physical activity rather than specific domains. For example, work- or travel-related physical activity tends to be involuntary, whereas leisure-time exercise is more voluntary and modifiable.

Based on the current research status and existing literature gaps, further investigation is urgently needed regarding factors such as exercise intensity, duration, and metabolic equivalents of tasks (METs). Therefore, this study aims to explore the association between physical exercise and OSA symptoms, as well as the relationships of exercise intensity, duration, and METs with OSA symptoms. The ultimate goal is to propose evidence-based exercise recommendations and provide a scientific basis for developing effective interventions to improve population health.

## 2. Methods and materials

### 2.1. Study population

All data for this study were sourced from the National Health and Nutrition Examination Survey (NHANES) dataset. NHANES is a nationally representative sample survey designed to collect information on the health and nutritional status of the United States population.^[18]^ The NHANES dataset is publicly available and does not require further ethical review board approval. Detailed information regarding data collection or other relevant details can be accessed on the NHANES website (https://wwwn.cdc.gov/nchs/nhanes/Default.aspx), which provides comprehensive information.

**Figure 1.**
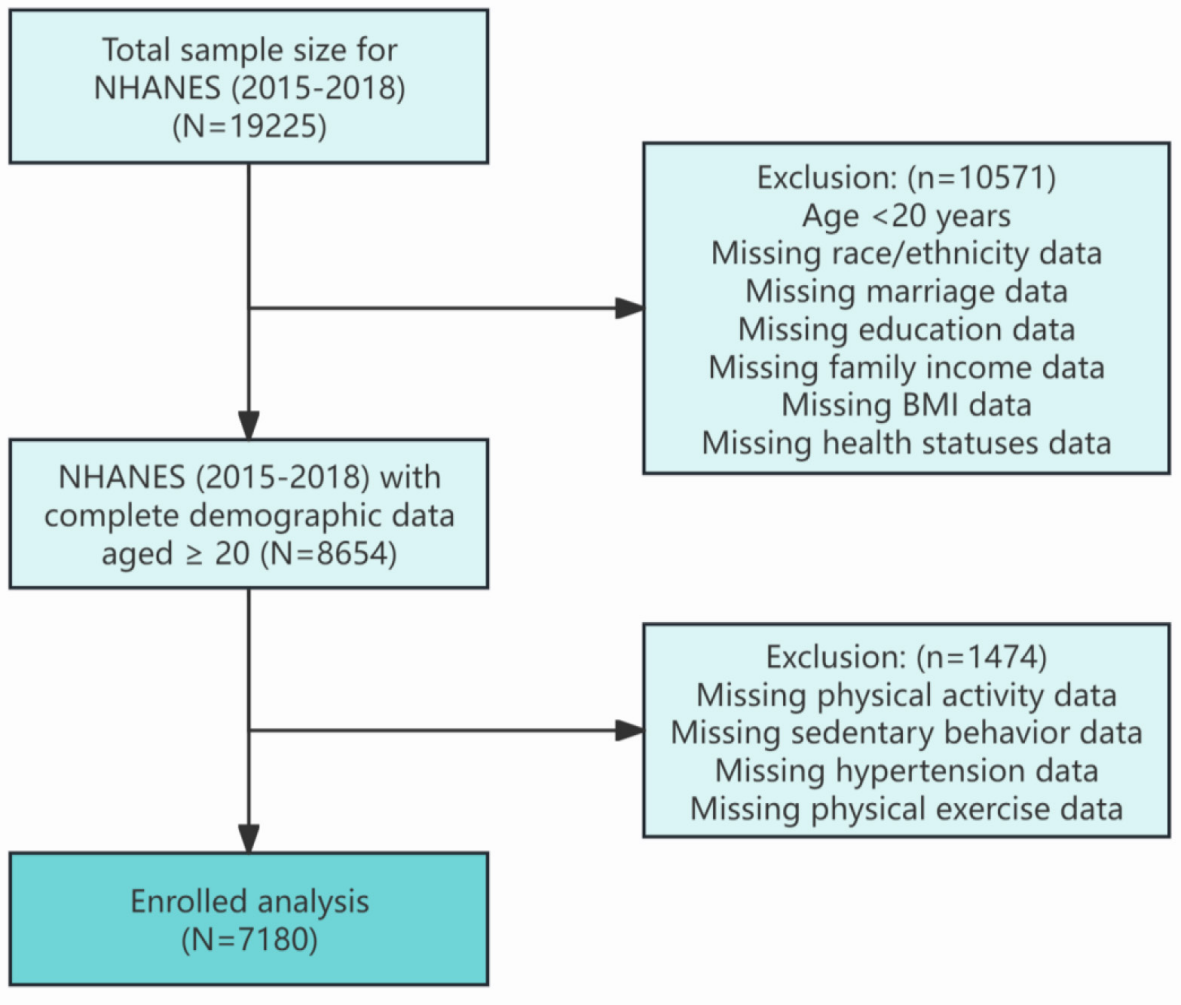
The data filtering process. BMI = body mass index, N/n – number of participants, NHANES = National Health and Nutrition Examination Survey.

A total of 19,225 individuals participated in the NHANES survey from 2015 to 2018. After excluding participants with missing data and those not meeting the inclusion criteria, 7180 individuals were finally included in the analyses. The detailed data screening process is presented in Figure 1.

### 2.2. Physical exercise assessment

Physical activity levels in the NHANES database were assessed using the Global Physical Activity Questionnaire (GPAQ). The GPAQ evaluated whether participants engaged in physical exercise during a typical week, as well as the intensity and duration of such exercise. In this study, the intensity of physical exercise was categorized into 3 levels: no physical exercise, moderate intensity, and high intensity.^[19]^ The GPAQ assessed various types of physical activity among participants, including occupational physical activity (e.g., heavy lifting), commuting physical activity (e.g., walking or cycling to work), physical exercise (e.g., ball games), and sedentary behavior (e.g., watching television, using the Internet). In this study, we used the more controllable physical exercise (e.g., ball games) from the NHANES database as the exposure variable. Exercise MET scores were calculated according to criteria recommended by the NHANES dataset: 1 minute of moderate-intensity exercise corresponded to 4 METs, and 1 minute of vigorous-intensity exercise corresponded to 8 METs.Accordingly, total weekly physical activity METs for each participant were computed as:


$$\begin{array}{cc} Total\;weekly\;METs=\left(minutes\;of\;moderate-intensity\;exercise\times 4\right)\\ & +\left(minutes\;of\;vigorous-intensity\;exercise\times 8\right). \end{array}$$

### 2.3. OSA symptoms assessment

In this study, OSA symptoms were assessed based on participants’ self-reported responses to sleep-related questions in the NHANES dataset. Referring to previous research,^[10]^ participants were defined as having OSA symptoms if they answered “yes” to at least one of the following 3 NHANES questions: experiencing excessive daytime sleepiness despite having at least 7 hours of sleep per night, reported to occur 16 to 30 times; having episodes of wheezing, gasping, or stopping breathing 3 or more times per week; and snoring 3 or more times per week.

### 2.4. Covariates

Drawing on previous research,^[20]^ we incorporated potential variables related to physical exercise and OSA symptoms as covariates. Among these, demographic variables included gender, age, race, education level, marital status, and income-to-poverty ratio. Age was categorized into 3 groups: 20 to 39, 40 to 59, and 60 and above. Race was classified as Hispanic, non-Hispanic White, non-Hispanic Black, non-Hispanic Asian, and other races.^[19]^ Education level was divided into less than high school and high school or above. Marital status was categorized as living with a partner, married but living alone (e.g., widowed, divorced, or separated), and never married. The income-to-poverty ratio was calculated by dividing household (or individual) income by the poverty threshold for the survey year. In this study, the poverty ratio was used to define 2 income conditions: low income (< 1.3) and moderate income (≥ 1.3).^[21]^ Additionally, other relevant variables included health status, work-related physical activity, transportation-related physical activity, and sedentary behavior. Health status was rated as excellent, very good, good, fair, or poor. Physical activity and sedentary behavior were assessed through questionnaires. In addition, body mass index (BMI) and hypertension were both included as key potential confounders for adjustment, given their close associations with both physical activity and OSA symptoms. BMI was categorized into underweight (≤ 18.9 kg/m^2^), normal weight (19.0–24.9 kg/m^2^), overweight (25.0–29.9 kg/m^2^), and obese (≥ 30.0 kg/m^2^).^[22]^ Hypertension was measured and diagnosed by qualified physicians.

### 2.5. Statistical analysis

We utilized Microsoft Excel 2010 to extract and merge the raw data, excluding items with missing or irrelevant (refused, unknown) entries. Missing data were handled using multiple imputation to minimize selection bias. All statistical analyses were performed among participants with complete data for key variables. The database included adults aged 20 years and above with complete information. This study employed sampling weights recommended by NHANES, which were derived from a stratified multistage probability sampling design. All analyses accounted for sample weights, clustering, and stratification to meet the analytical requirements of NHANES data and generate nationally representative sample statistics. Based on the research objectives, we conducted significance tests to compare the differences in covariates between the “OSA symptoms group” and the “non-OSA symptoms group.” For continuous variables, we employed the rank-sum test, while for categorical variables, we used the chi-square test. The relationship between physical exercise and OSA symptoms was analyzed using a binary logistic regression model. All data analyses were performed using Statistical Package for the Social Sciences 26.0 (IBM Corporation, Armonk), with *P* values < .05 considered statistically significant (2-tailed test).

In this study, all covariates (*P *< .05) demonstrated statistical significance in the univariate analysis, except for the income-to-poverty ratio (*P *= .309). When analyzing the relationship between physical exercise and OSA symptoms using the binary logistic regression model, physical exercise was set as the independent variable and OSA symptoms as the dependent variable. To exclude the influence of covariates, we conducted both univariate analysis (without adjusting for any covariates) and multivariate analysis (adjusting for all covariates, including gender, age, race, education level, marital status, income to poverty, BMI, health status, work-related physical activity, transportation-related physical activity, sedentary behavior, and hypertension).

The restricted cubic spline (RCS) curves used in this study were generated using R software (version 4.3.3). These curves illustrated the nonlinear relationship between the dependent and independent variables. The horizontal axis represented the duration of physical exercise, while the vertical axis displayed the odds ratio (OR) and its 95% confidence interval (CI) for the association between the duration of physical exercise and OSA symptoms. A *P* value < .05 was considered statistically significant. RCS analysis was performed with 3 knots placed at the 5th, 50th, and 95th percentiles using the percentile method. The median value of the exposure variable was used as the reference. Covariates adjusted for in the model included: gender, age, race, education level, marital status, income to poverty, BMI, health status, work-related physical activity, transportation-related physical activity, sedentary behavior, and hypertension. The overall association *P*-value and the nonlinearity test *P* value were reported. All RCS analyses were conducted accounting for the complex NHANES sampling design, incorporating sampling weights, stratification, and primary sampling units.

To explore the threshold effect between the duration of physical exercise and OSA symptoms, we conducted a threshold effect analysis. The segmented package in R software (version 4.3.3) was used to identify potential breakpoints (i.e., thresholds) in the relationship between the independent and dependent variables.^[23]^ Linear relationships before and after the breakpoints were modeled and analyzed separately. Threshold analysis was performed using a 2-segment linear regression model to identify the breakpoint. The breakpoint was estimated via grid search, and its 95% CI was derived using the bootstrap method with 1000 resamplings. Before analysis, the exposure variable was standardized or scaled in original units to ensure a consistent measurement scale. This approach allowed us to gain a deeper understanding of the complex relationship patterns between the 2 variables, determine the optimal threshold, and clarify the threshold effect relationship between them.

## 3. Results

### 3.1. Demographic characteristics

This study encompassed 7180 adults aged 20 years and above who participated in the NHANES from 2015 to 2018. Participants provided data on physical exercise, OSA symptoms, and demographic information. The study findings revealed significant differences between the OSA symptoms group and the non-OSA symptoms group in terms of gender, age, race, education level, marital status, BMI, health status, work-related physical activity, transportation-related physical activity, sedentary behavior, hypertension, and physical exercise, with these differences being statistically significant (*P* < .05). No significant difference was observed in the income-to-poverty ratio (*P* = .309). Notably, among these differences, the between-group variations in BMI, hypertension, health status, and physical exercise possess certain clinical relevance, suggesting that these factors may be meaningfully associated with OSA symptoms. (See Table 1)

**Table 1:** Characteristics of the study population (N = 7180).

Variables	Total (n = 7180)	Non-OSA symptoms (n = 3419)	OSA symptoms (n = 3761)	Statistic	*P*
Age	49.45 ± 17.48	47.43 ± 18.41	51.28 ± 16.37	Z = -9.33	< .001
Gender				χ^2^ = 43.36	< .001
Male	3535 (49.23)	1544 (45.16)	1991 (52.94)		
Female	3645 (50.77)	1875 (54.84)	1770 (47.06)		
Race				χ^2^ = 41.40	< .001
Hispanic	1877 (26.14)	791 (23.14)	1086 (28.88)		
Non-Hispanic White	2567 (35.75)	1251 (36.59)	1316 (34.99)		
Non-Hispanic Black	1532 (21.34)	734 (21.47)	798 (21.22)		
Non-Hispanic Asian	886 (12.34)	482 (14.10)	404 (10.74)		
Other	318 (4.43)	161 (4.71)	157 (4.17)		
Education				χ^2^ = 8.74	.013
Below high school	1412 (19.67)	627 (18.34)	785 (20.87)		
High school graduate	1623 (22.60)	765 (22.37)	858 (22.81)		
Post high school	4145 (57.73)	2027 (59.29)	2118 (56.31)		
Marital Statues				χ^2^ = 71.78	< .001
Cohabitation	4503 (62.72)	2027 (59.29)	2476 (65.83)		
Married living alone	1403 (19.54)	649 (18.98)	754 (20.05)		
Not married	1274 (17.74)	743 (21.73)	531 (14.12)		
Income to Poverty				χ^2^ = 1.03	.309
Impoverished	2097 (29.21)	979 (28.63)	1118 (29.73)		
Moderate income	5083 (70.79)	2440 (71.37)	2643 (70.27)		
BMI				χ^2^ = 364.03	< .001
Underweight	160 (2.23)	120 (3.51)	40 (1.06)		
Normal weight	1782 (24.82)	1115 (32.61)	667 (17.73)		
Overweight	2293 (31.94)	1119 (32.73)	1174 (31.22)		
Obese	2945 (41.02)	1065 (31.15)	1880 (49.99)		
Current Health Status				χ^2^ = 172.45	< .001
Excellent	633 (8.82)	383 (11.20)	250 (6.65)		
Very good	1839 (25.61)	1015 (29.69)	824 (21.91)		
Good	3028 (42.17)	1399 (40.92)	1629 (43.31)		
Fair	1462 (20.36)	563 (16.47)	899 (23.90)		
Poor	218 (3.04)	59 (1.73)	159 (4.23)		
WPA				χ^2^ = 9.81	.002
No	3274 (45.60)	1493 (43.67)	1781 (47.35)		
Yes	3906 (54.40)	1926 (56.33)	1980 (52.65)		
CPA				χ^2^ = 13.33	< .001
No	1620 (22.56)	836 (24.45)	784 (20.85)		
Yes	5560 (77.44)	2583 (75.55)	2977 (79.15)		
Physical Exercise				χ^2^ = 49.42	< .001
No	3622 (50.45)	1576 (46.10)	2046 (54.40)		
Yes	3558 (49.55)	1843 (53.90)	1715 (45.60)		
Sedentary Behavior				χ^2^ = 11.03	< .001
< 600	5956 (82.95)	2889 (84.50)	3067 (81.55)		
≥ 600	1224 (17.05)	530 (15.50)	694 (18.45)		
Hypertension				χ^2^ = 122.40	< .001
Yes	2578 (35.91)	1003 (29.34)	1575 (41.88)		
No	4602 (64.09)	2416 (70.66)	2186 (58.12)		

Z = Mann-Whitney test, χ^2^ = Chi-square test.

BMI = body mass index, CPA = commuting physical activity, N/n – number of participants, OSA = obstructive sleep apnea, WPA = work physical activity.

### 3.2. Association between physical exercise and OSA symptoms

In the current logistic regression analysis, the univariate analysis (without adjusting for any covariates) indicated an OR of 0.72 (95% CI: 0.65–0.79) for the association between physical exercise and OSA symptoms. The multivariate analysis (adjusting for variables such as gender, age, race, education level, marital status, BMI, income to poverty, health status, work-related physical activity, transportation-related physical activity, sedentary behavior, and hypertension) revealed an OR of 0.84 (95% CI: 0.76–0.93). Our findings indicated that physical exercise was inversely associated with OSA symptoms (*P* < .05). (See Table 2)

**Table 2:** Binary logistic regression analysis of physical exercise and OSA symptoms.

Variables	Univariate analysis					Multivariate analysis				
	*β*	SE	Z	*P*	OR (95% CI)	*β*	SE	Z	*P*	OR (95% CI)
Exercise										
No					1.00 (reference)					1.00 (reference)
Yes	−0.33	0.05	−7.02	< .001	0.72 (0.65–0.79)	−0.17	0.05	−3.23	.001	0.84 (0.76–0.93)

Univariate analysis: without adjusting for any covariates; multivariate analysis: adjusting for all covariates.

CI = confidence interval, OR = odds ratio, OSA = obstructive sleep apnea, SE = standard error.

### 3.3. Association between physical exercise intensity and OSA symptoms

In the binary logistic regression analysis of physical exercise intensity and OSA symptoms, we used the group with no physical exercise as the reference. The univariate analysis (without adjusting for any covariates) revealed an OR of 0.86 (95% CI: 0.77–0.96) for the association between moderate-intensity physical exercise and OSA symptoms, and an OR of 0.60 (95% CI: 0.54–0.67) for the association between high-intensity physical exercise and OSA symptoms. The multivariate analysis (adjusting for variables such as gender, age, race, education level, marital status, BMI, income to poverty, health status, work-related physical activity, transportation-related physical activity, sedentary behavior, and hypertension) showed an OR of 0.89 (95% CI: 0.79–1.00) for the association between moderate-intensity physical exercise and OSA symptoms, and an OR of 0.79 (95% CI: 0.69–0.90) for the association between high-intensity physical exercise and OSA symptoms. The results indicated that, compared with the inactive group, individuals engaging in moderate-intensity physical activity weekly had a 0 to 21% lower odds of OSA symptoms, with the difference being marginally significant (*P* = .059); while those engaging in high-intensity physical exercise per week had a 10 to 31% reduced probability of developing OSA symptoms (*P* < .05). (See Table 3)

**Table 3:** Binary logistic regression analysis of physical exercise intensity and OSA symptoms.

Variables	Univariate analysis					Multivariate analysis				
	*β*	SE	Z	*P*	OR (95% CI)	*β*	SE	Z	*P*	OR (95% CI)
Intensity										
No					1.00 (reference)					1.00 (reference)
Moderate	−0.15	0.06	−2.59	.010	0.86 (0.77–0.96)	−0.12	0.06	−1.88	.059	0.89 (0.79–1.00)
Dramatic	−0.51	0.06	−8.76	< .001	0.60 (0.54–0.67)	−0.24	0.07	−3.52	< .001	0.79 (0.69–0.90)

Univariate analysis: without adjusting for any covariates; multivariate analysis: adjusting for all covariates.

CI = confidence interval, OR = odds ratio, OSA = obstructive sleep apnea, SE = standard error.

### 3.4. Association between physical exercise duration and OSA symptoms

**Figure 2.**
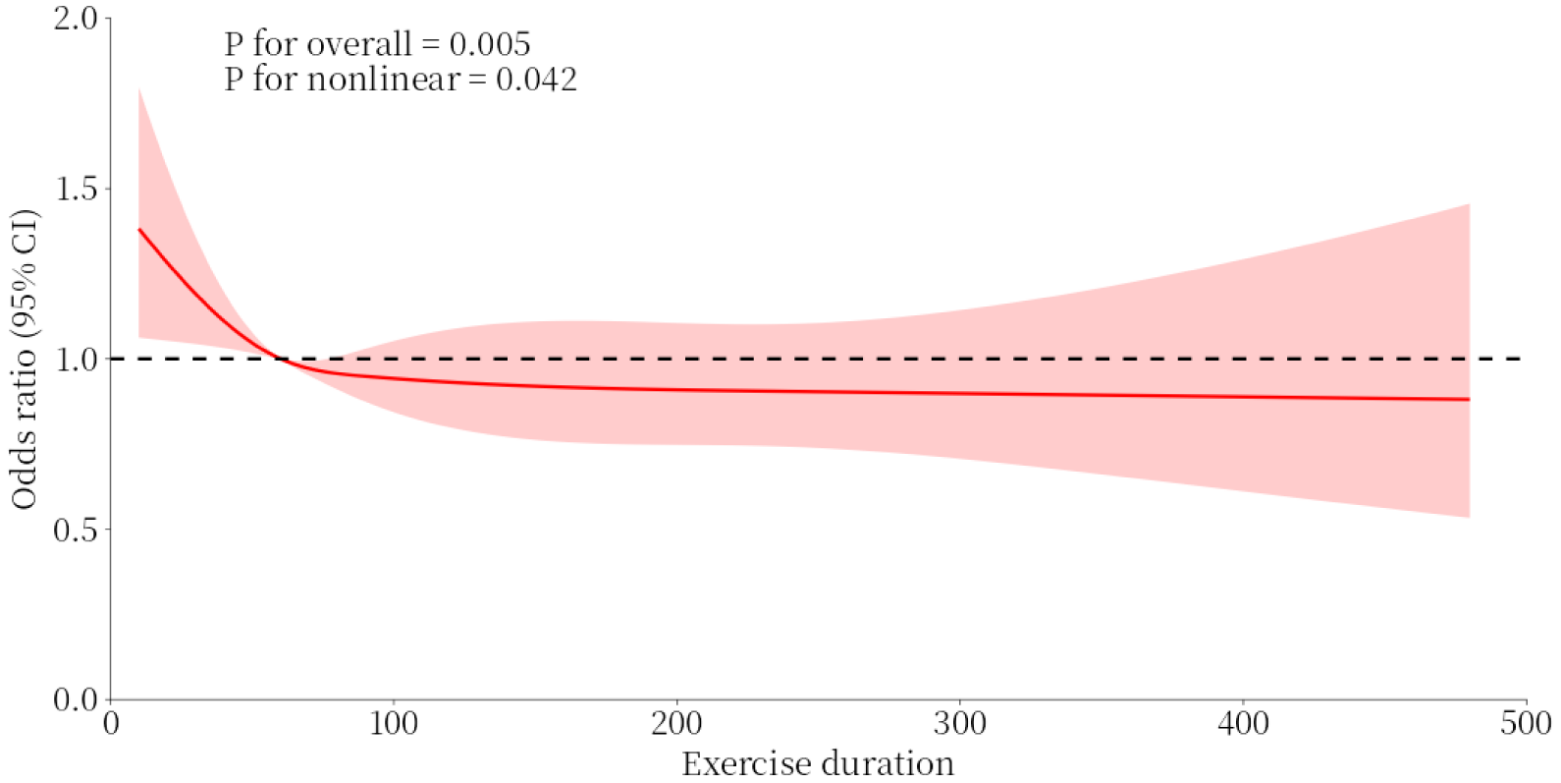
RCS curves of the association between physical exercise duration and OSA symptoms. The solid red line represents the adjusted OR for OSA symptoms across varying exercise durations, with the shaded red area indicating the corresponding 95% CIs. The dashed black line at OR = 1 serves as the reference line, where values below indicate reduced risk and values above indicate increased risk. Statistical tests revealed a significant overall association (*P* overall = .005) and a confirmed nonlinear relationship (*P* nonlinear = .042), indicating a gradual, progressive association between exercise duration and OSA symptom risk rather than a clear threshold effect. CI = confidence interval, RCS = restricted cubic spline, OR = odds ratio, OSA = obstructive sleep apnea.

As shown in Figure 2, results from RCS analysis demonstrated a significant overall association between exercise duration and OSA symptoms (*P* for overall < .05) with a pronounced nonlinear pattern (*P* for nonlinear < .05). The median value of exercise duration was used as the reference, corresponding to an OR of 1.0 (dashed line in the figure). The model was fully adjusted for confounders including gender, age, race, education level, marital status, BMI, income to poverty, health status, work-related physical activity, transportation-related physical activity, sedentary behavior, and hypertension, and all analyses were weighted to account for the complex sampling design of NHANES. The curve showed a gradual decrease in risk with increasing exercise duration, with the OR dropping from above 1 to below 1 at approximately 70 minutes, which differs from the breakpoint estimated by threshold analysis. As RCS analysis was designed to explore the continuous dose-response relationship while threshold analysis focused on statistical testing of potential effect change points, the discrepancy in results is attributable to the distinct analytical purposes of the 2 methods.

**Figure 3.**
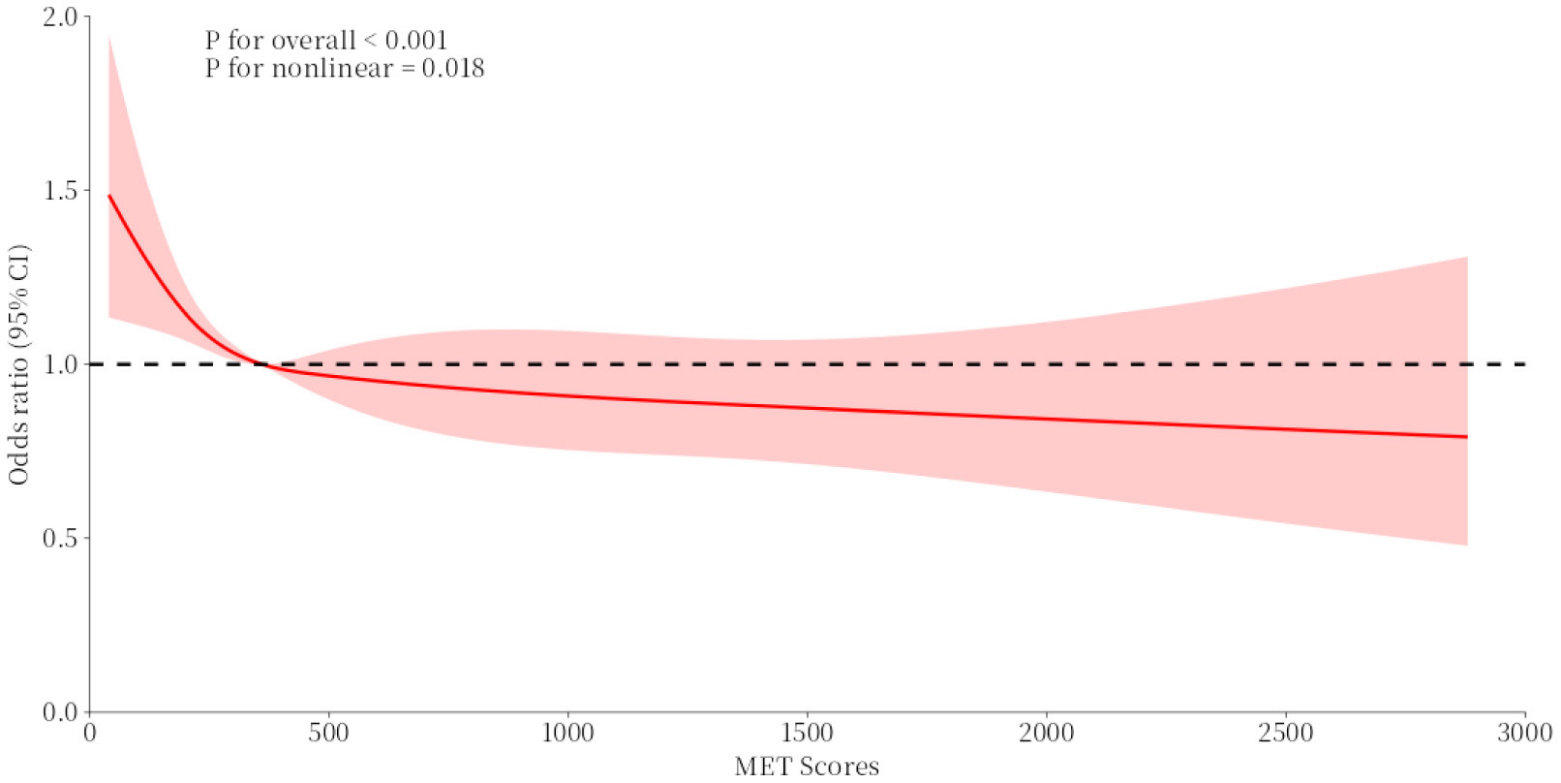
RCS curves of the association between physical exercise MET and OSA symptoms. The solid red line represents the adjusted OR for OSA symptoms across varying exercise MET values, with the shaded red area indicating the corresponding 95% CIs. The dashed black line at OR = 1 serves as the reference line, where values below indicate reduced risk and values above indicate increased risk. Statistical tests revealed a significant overall association (*P* overall < .001) and a confirmed nonlinear relationship (*P* nonlinear = .018), consistent with a gradual change trend in the association between exercise MET and OSA symptom risk. CI = confidence interval, MET = metabolic equivalent of task, RCS = restricted cubic spline, OR = odds ratio, OSA = obstructive sleep apnea.

As shown in Figure 3, RCS analysis revealed a significant overall association between METs and OSA symptoms (*P* overall < .05) with a significant nonlinear trend (*P* nonlinear < .05). The curve demonstrated a gradual, progressive reduction in risk with increasing exercise volume, and the OR decreased from above 1 to below 1 at approximately 400 MET-minutes without an obvious cutoff point.

To more accurately understand the nonlinear relationship between physical exercise duration or MET and OSA symptoms, and to identify the turning point in the relationship between these 2 variables, we conducted a threshold effect analysis.

**Figure 4.**
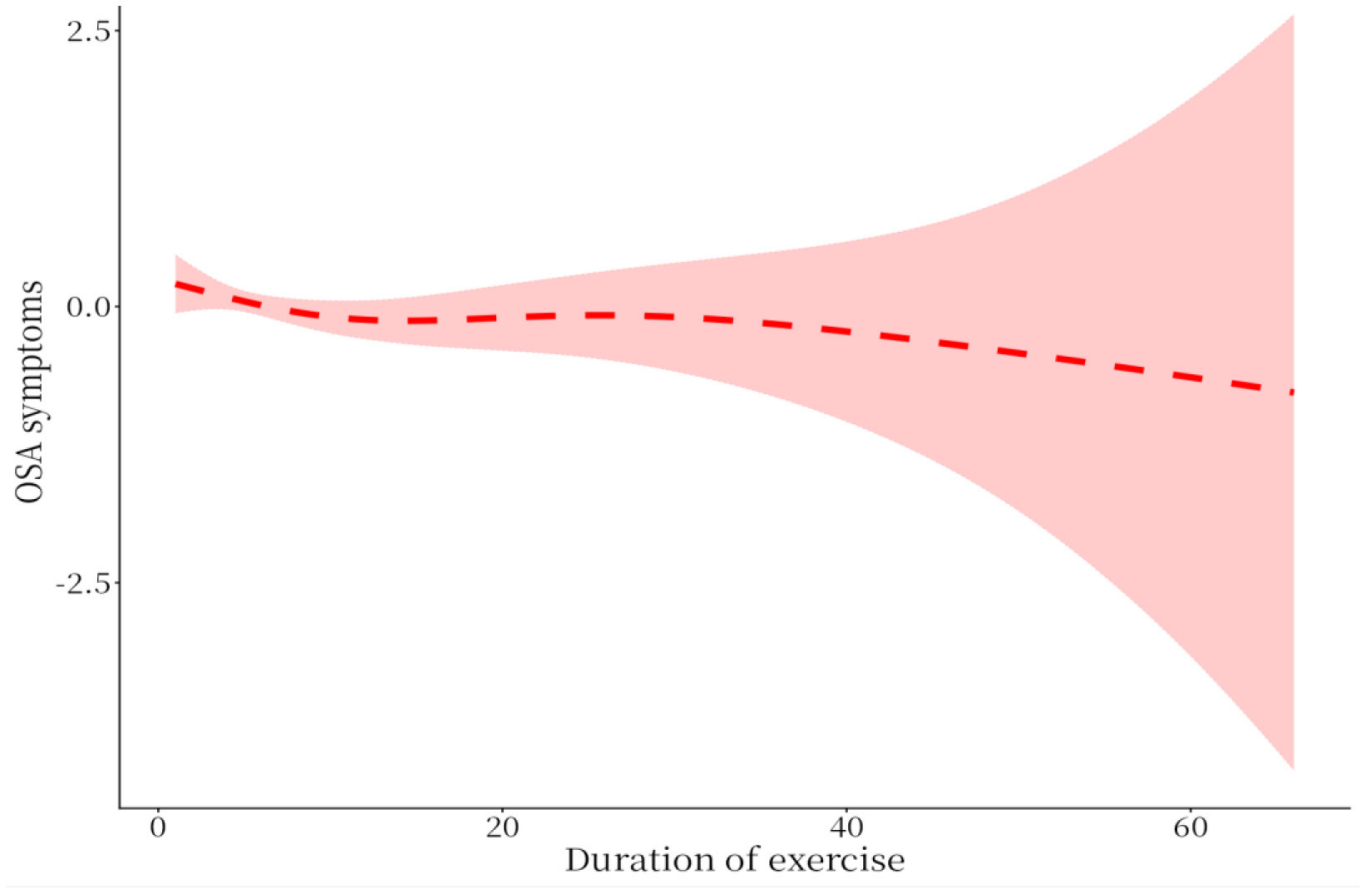
Threshold analysis of the association between physical exercise duration and OSA symptoms. The red dashed line represents the estimated association between exercise duration and OSA symptoms, with the pink shaded area indicating the 95% CI. The graphical trend reflects a gradual variation in the association rather than a distinct, definitive threshold. CI = confidence interval, OSA = obstructive sleep apnea.

As shown in Table 4 and Figure 4, threshold analysis was performed per 10-minute increment in exercise duration. The standard linear model yielded an OR of 0.99 (95% CI: 0.98–1.00, *P* < .05). A 2-segment model suggested a potential breakpoint at 75 minutes, with an OR of 0.93 (95% CI: 0.89–0.98, *P* < .05) for duration < 75 minutes, while the association was nearly null (OR = 1.00, 95% CI: 0.98–1.01, *P* = .718) for duration ≥ 75 minutes. The likelihood ratio test was significant (*P* < .05), though the effect size was modest beyond the threshold.

**Table 4:** Results of threshold effect analysis of physical exercise duration and OSA symptoms.

Outcome	Effect	*P*
Model 1 Fitting model by standard linear regression	0.99 (0.98–1.00)	.010
Model 2 Fitting model by 2-piecewise linear regression		
Inflection point	7.5	
< 75.00	0.93 (0.89–0.98)	.004
≥ 75.00	1.00 (0.98–1.01)	.718
*P* for likelihood test		.023

OSA = obstructive sleep apnea.

**Figure 5.**
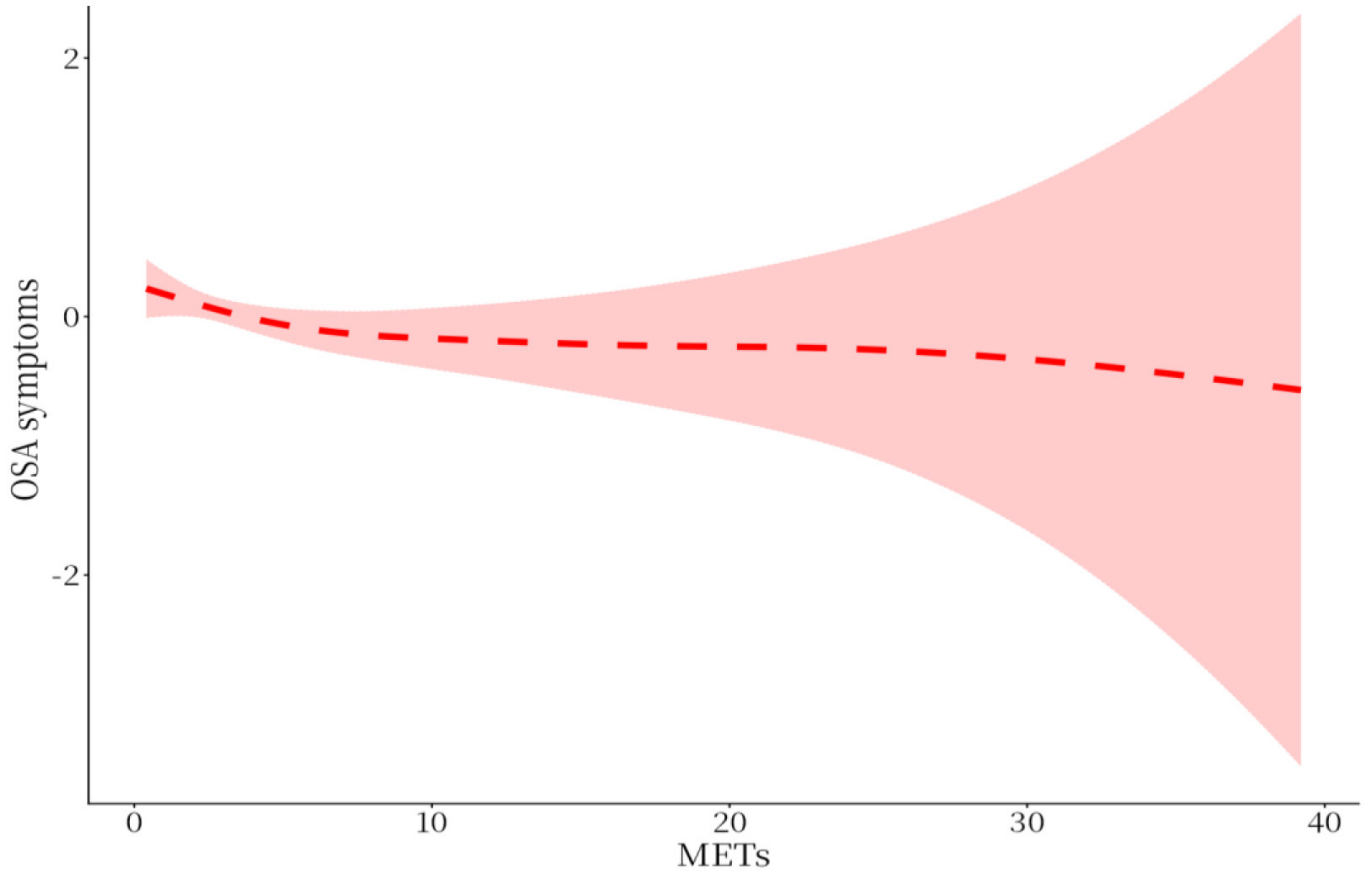
Threshold analysis of the association between physical exercise MET and OSA symptoms. The red dashed line represents the estimated association between exercise MET and OSA symptoms, with the pink shaded area indicating the 95% CI. Consistent with other figures, the trend suggests a gradual change in the association without a clear cutoff point. CI = confidence interval, MET = metabolic equivalent of task, OSA = obstructive sleep apnea.

As shown in Table 5 and Figure 5, threshold analysis was conducted per 100 METs. The linear model showed an OR of 0.97 (95% CI: 0.96–0.99, *P* < .05). A 2-segment model indicated a potential breakpoint at 320 METs, with an OR of 0.87 (95% CI: 0.76–1.00, *P* = .054) below this level, and a nearly null association (OR = 0.99, 95% CI: 0.97–1.01, *P* = .365) at or above 320 METs. Although the likelihood ratio test was significant (*P* < .05), the effect size was relatively small beyond the threshold.

**Table 5:** Results of threshold effect analysis of physical exercise MET and OSA symptoms.

Outcome	Effect	*P*
Model 1 Fitting model by standard linear regression	0.97 (0.96–0.99)	< .001
Model 2 Fitting model by 2-piecewise linear regression		
Inflection point	3.20	
< 320.00	0.87 (0.76–1.00)	.054
≥ 320.00	0.99 (0.97–1.01)	.365
*P* for likelihood test		.015

MET = metabolic equivalent of task, OSA = obstructive sleep apnea.

## 4. Discussions

Previous studies have indicated a negative correlation between physical activity levels and the prevalence of OSA symptoms.^[24,25]^ Conversely, some research has suggested no significant association between physical activity and OSA symptoms.^[26]^ The present study confirmed that physical exercise was inversely associated with OSA symptoms, which is consistent with previous findings. Furthermore, our research reveals that high-intensityexercise exhibits a more pronounced negative association with OSA symptoms compared to moderate-intensity exercise.

From the perspective of airway structure and function, insufficient physical exercise weakens the muscles surrounding the upper airway, exacerbating relaxation during sleep and increasing the likelihood of airway collapse and narrowing.^[27]^ Additionally, lack of physical exercise contributes to obesity, leading to the accumulation of fat around the upper airway, such as in the neck, which compresses the pharyngeal cavity and increases the risk of airway obstruction.^[28,29]^ Respiratory regulation is also compromised, as reduced exercise diminishes respiratory muscle strength, affecting the regulation of breathing depth and frequency, resulting in unstable respiratory control during sleep and an increased risk of apnea or hypoventilation, thereby triggering OSA symptoms.^[30]^ Moreover, inadequate exercise impedes venous return, causing fluid accumulation in the distal vessels and interstitial spaces of the lower extremities. This accumulated fluid migrates to the neck during sleep, increasing upper airway resistance and contributing to the development of OSA symptoms.^[17]^

Metabolic and endocrine disturbances also play significant roles. Lack of exercise enhances insulin resistance, triggering systemic inflammatory responses that lead to airway inflammation and edema. It also affects the secretion of hormones such as leptin and adiponectin, increasing appetite, promoting fat accumulation, and disrupting metabolism, thereby elevating the risk of OSA symptoms.^[31]^ Furthermore, insufficient physical exercise alters sleep architecture and quality, leading to increased light sleep and reduced deep sleep, along with impaired respiratory regulation.^[32]^ It also causes sleep fragmentation, disrupting sleep cycles and respiratory rhythms, preventing the respiratory center from adequately resting and regulating, and inducing hormonal changes that further affect metabolic and respiratory functions, collectively increasing the prevalence of OSA symptoms.^[33]^

From a physiological adaptation standpoint, moderate weekly exercise of around 75 minutes may provide adequate systemic stimulation to improve cardiopulmonary function and circulation efficiency. Appropriate physical activity is conducive to maintaining upper airway muscle function and relieving nocturnal airway collapse risk.^[34,35]^ Meanwhile, moderate exercise can regulate metabolism and body composition, reduce neck fat deposition, and mitigate upper airway compression. Consistent with prior research, excessive physical activity does not always confer extra sleep benefits.^[32]^ The approximate turning point of 75 minutes observed in our study reflects a moderate activity range, which is in line with existing evidence. When cumulative exercise METs approach 320, moderate-to-vigorous activity may mildly modulate neuroendocrine and autonomic nervous function, helping stabilize sleep rhythms and reduce sleep-disordered breathing events.^[36]^ Exercise-related metabolic regulation can optimize systemic metabolic status and respiratory regulation, which may partly explain the favorable correlation between proper physical activity and reduced OSA symptom burden.^[37,38]^ All observed statistical turning points in this analysis should be regarded as reference trends rather than definitive critical thresholds.

## 5. Limitations and perspectives of this study

This study further elucidates the association between physical exercise and OSA symptoms, demonstrating innovation and practical significance. However, several limitations persist: in the present study, both physical exercise and OSA symptoms were assessed based on self-reports by participants, which may lead to under- or overestimation of actual physical activity levels and OSA symptoms; this study adopted a cross-sectional design, which precludes causal inference; the inclusion of covariates in this study was not comprehensive enough to exclude the influence of all potentially relevant variables.

Future research should endeavor to overcome these limitations. Moreover, exploring the associations between other forms of physical activity (e.g., labor, commuting) and OSA symptoms, as well as delving deeper into the underlying mechanisms of the relationship between physical activity and OSA symptoms, are crucial. These studies will contribute to the treatment and prevention of OSA symptoms, thereby promoting human health.

## 6. Conclusion

The results of this study indicate a negative correlation between physical exercise and OSA symptoms, with a more pronounced negative association observed for high-intensity physical exercise. This observational study identified a nonlinear association between physical activity levels and OSA symptoms. The correlation pattern varied across different exercise durations and cumulative MET values, with subtle shifts in the association trend observed at certain activity ranges. Under the premise of maintaining overall health, appropriately engaging in moderate-to-vigorous physical activity weekly and achieving corresponding activity levels may help alleviate OSA-related symptoms. Future population-based intervention studies can further explore the practical value of these activity levels in improving sleep breathing outcomes.

## Author Contributions

**Conceptualization:** Yan Sun, Kexin Zhu.

**Data curation:** Yan Sun.

**Formal analysis:** Yan Sun, Kexin Zhu.

**Investigation:** Yan Sun.

**Methodology:** Yan Sun, Kexin Zhu.

**Project administration:** Kexin Zhu.

**Resources:** Kexin Zhu.

**Software:** Yan Sun.

**Supervision:** Kexin Zhu.

**Validation:** Kexin Zhu.

**Visualization:** Yan Sun.

**Writing – original draft:** Yan Sun, Kexin Zhu.

**Writing – review & editing:** Yan Sun, Kexin Zhu.
